# Realist review protocol for understanding young people’s experiences of engaging with police-mental health practitioner collaboration in emergency responses to mental health crises

**DOI:** 10.1186/s13643-025-02882-4

**Published:** 2025-09-24

**Authors:** Sarah Parry, Lucy Oakes, Zarah Eve, Stephen Edwards, Fiona Lobban, Prathiba Chitsabesan, Geoff Wong

**Affiliations:** 1 Pennine Care NHS Foundation Trust, 225 Old Street, Ashton-Under-Lyne, Lancashire, UK; 2 School of Health Sciences, The University of Manchester, Manchester, UK; 3 Department of Psychology, Manchester Metropolitan University, Manchester, M15 6GX UK; 4 Lancaster University Faculty of Health and Medicine, Lancaster University, Lancaster, UK; 5 Medical Sciences Division, Nuffield Department of Primary Care Health Sciences, The University of Oxford, Oxford, UK; 6 Division of Psychiatry, University College London, London, UK

**Keywords:** Children, Young people, Mental health, Crisis, Police, Co-response, Emergency responder, Realist review, Protocol

## Abstract

**Background:**

Children and young people are facing increasing mental health challenges. Access to emergency mental health care for young people is under-researched and poorly understood. Police data indicates a rise in youth mental health emergency calls, but officers often feel unprepared to support young people in crisis. Mental health practitioners have the experience and training to provide helpful support to young people in crisis, although the availability of mental health services for young people can be limited during evenings and weekends, especially in rural areas. We know that children and young people can benefit when police and mental health services work together. However, we need to better understand the full range of impacts of joint responses for young people and their families and how these impacts are generated. Joint emergency response is a complex intervention, and a realist synthesis was chosen as it can make sense of such interventions. Therefore, this realist synthesis aims to develop a programme theory of the underlying generative mechanisms by which, and contexts within which, emergency responders collaborate and co-respond to support young people experiencing a mental health crisis.

**Methods and analysis:**

We will follow five steps to undertake the realist review: (1) Define the review scope, (2) develop initial programme theories, (3) conduct an evidence search, (4) select and appraise evidence, and (5) extract and synthesise data. Embase, CINAHL, Social Policy and Practice, MEDLINE, PsycINFO, and AMED databases will be searched up to June 2024, supplementing searches with citation tracking, grey literature, relevant NHS England guidance, and practitioner interpretation workshops. Data selection will be based on relevance and richness. Data will be extracted and synthesised iteratively, and causal links between contexts, mechanisms, and outcomes will be illuminated in the process. The results will be conducted and reported according to the Realist and Meta-narrative Evidence Syntheses: Evolving Standards (RAMESES) quality and publication standards.

**Collaboration and dissemination:**

Findings will be disseminated to the research community through conference presentations and a peer-reviewed journal article. We will work with healthcare and police organisations, as well as professional and expert-by-experience stakeholder groups, including commissioners, to develop a strategy for far-reaching dissemination with impact to share findings across a range of audiences.

**Discussion:**

This study will develop a programme theory regarding how emergency responders collaborate to support young people experiencing mental health crises. Findings will inform future practices, aiming to improve collaborative responses and outcomes across youth contexts.

**Systematic review registration:**

PROSPERO CRD42024542081.

**Supplementary Information:**

The online version contains supplementary material available at 10.1186/s13643-025-02882-4.

## Strengths and limitations

- The realist review addresses a recognised knowledge gap in crisis services in the UK for children, young people, and families.
- The focus upon international literature with children, young people, and young/emerging adults (up to and including age 25) reduces the likelihood of findings prioritising only adult or all-age services.
- This review’s novel contribution lies in combining published literature, grey literature, and anonymised stakeholder workshop transcripts — specifically focusing on the collaboration between police officers and NHS practitioners in crisis pathways for children and young people.
- The review will be limited by only including English language studies, which is necessary due to the resources available to the team.

## Background and rationale

Children and young people are experiencing increasing challenges to their mental health, with services ‘constantly firefighting’ to meet demand [1]. Pathways to mental health care for young people are under-researched [2] and poorly understood. Currently, the NHS is not reaching targets to increase access and reduce waiting times for young people’s mental health services [3]. Data collected by Greater Manchester Police (GMP) shows youth mental health care plans developed by police officers attending to 999 calls increased by 14.2% between 2021 and 2022, demonstrating an overall increase in the number and severity of youth mental health call-outs during this period. The increasing prevalence of police attendances for mental health crises in the UK can be attributed to multiple factors, including rising demand for mental health services, reductions in community-based crisis care, and a growing recognition of mental health as a public health issue. Policy initiatives, such as the Crisis Care Concordat, have also reinforced the role of police in emergency mental health responses, often filling gaps left by stretched health services. Police officers often feel ill-equipped to provide mental health support, especially to children and young people experiencing a mental health crisis [4]. Therefore, there is a clinical need to improve access to and experiences of emergency responses and crisis care for young people experiencing a mental health crisis. A mental health crisis refers to a situation in which an individual experiences acute psychological distress, an inability to cope, or poses a risk to themselves or others. This can include suicidal ideation, severe anxiety, psychosis, or extreme emotional distress requiring urgent intervention [5]. In the context of this study, the focus will be on crises that necessitate emergency responses from co-responding police officers and mental health practitioners.

Specific suggestions for improvements to crisis services have already been made. For instance, Wolff et al. (2023) suggest that creating a more welcoming and less clinical atmosphere within emergency departments can alleviate anxiety and improve overall experiences [6]. Additionally, mapping the patient journey from the perspective of both patients and staff can identify bottlenecks and areas for improvement in emergency departments [7]. The most recent systematic review of emergency mental healthcare for children and young people [8] emphasises the importance of comprehensive training for emergency responders. Training should focus on recognising and managing mental health crises with children and young people, aiming to equip staff with the skills necessary to provide appropriate initial care and referrals [9]. Based on a recent pilot in Greater Manchester, England, the involvement of specialised mental health professionals significantly improves the response to young people in crisis, offering targeted interventions, ensuring continuity of care, and reducing the likelihood of repeated crises [10]. According to Trainor and O’Connor (2023), integrating mental health services within emergency care frameworks is crucial [11]. This includes establishing clear protocols for the involvement of mental health professionals and creating pathways that facilitate swift and effective transitions from emergency care to long-term support. Currently, research indicates there can be potential risks when first responders attend to young people experiencing a mental health crisis, which may impact care outcomes. In some cases, efforts to ensure safety, such as encouraging individuals to leave their homes to facilitate police intervention under Sect. 136 of the Mental Health Act, may unintentionally contribute to feelings of distrust and discourage future help-seeking [12]. The quality of care provided by ambulance services can also be inconsistent, with some young people describing their experiences as inadequate or unhelpful [13]. Additionally, much like the police force, paramedics may not have specialised mental health training, which can affect their ability to manage self-harm and crisis situations effectively [14, 15]. The emotional demands of responding to mental health emergencies can also contribute to stress and burnout among first responders, potentially impacting their capacity to provide compassionate care [16]. Furthermore, emergency interventions, even when well-intentioned, may lead to feelings of stigma, fear, or distress among young people, potentially exacerbating their mental health difficulties [17]. To date, there is no overarching theory about how an integrated or collaborative approach to youth crisis support might work in different contexts to inform policy and practice. Consequently, this review aims to develop a programme theory to inform the development of an optimal integrated co-response between mental health practitioners and police officers to support young people experiencing mental health crises.

Throughout this review, we refer to ‘co-responders’ and the collaborative teams consisting of mental health professionals, paramedics, and police officers who work together in joining response efforts to mental health crises. Specifically, parademics are sometimes included as part of the emergency response teams, which can also involve police officers and mental health specialists. We use the term ‘first responders’ to refer to individuals who are the initial responders to emergency situations, including paramedics, police officers, and other emergency personnel, depending on jurisdiction. In this context, ‘emergency response’ refers to the entire process of addressing a mental health emergency, from the initial call to the final intervention, which may involve hospitalisation, intervention, or referral to other services.

Collaborations nationally, typically involving mental health services, police, and paramedics, have demonstrated that joint response approaches reduce the inappropriate use of mental health legislation and hospital admissions [18], increase engagement between patients and professionals [19], strengthen relationships between the police [20] and health services [21], and reduce costs to public services [22]. Recent analysis of personal accounts of service users indicates families generally feel safer and more supported when a co-response team is involved [19]. Combined responses from police officers, paramedics, and mental health professionals have been reported to be better equipped to manage mental health emergencies, with a more holistic and compassionate approach. Overall, improved communication and coordination among different services are beneficial, leading to timely and appropriate care for people in crisis. Moreover, the presence of mental health professionals within response teams helps to de-escalate situations that might otherwise lead to involuntary hospitalisation. Family members have also reported feeling reassured by the involvement of specialised mental health practitioners alongside emergency services, further suggesting co-responses are worthy of further exploration [23].

Co-response models seem to demonstrate benefits for young people experiencing mental health crises. The literature available on co-responses, predominantly from Australia, New Zealand, Canada, and the USA, highlights the efficacy and mechanisms underpinning these co-response models [24, 25]. A key benefit is the reduction in use of restrictive practices, which impact some communities disproportionately in the UK, particularly in relation to young Black men’s contact with the police [26]. In Canada, for example, Blais and Brisebois (2021) found co-responses led to more peaceful resolutions compared to traditional police responses [27]. Similarly, Lowder et al. (2024) reported co-response models in the USA resulted in fewer arrests and physical confrontations [20]. The emerging literature on co-responses also suggests improved mental health outcomes, as the presence of mental health professionals ensures people in need receive immediate psychological assessment and intervention. In Australia and New Zealand, co-response models have been found to facilitate quicker access to mental health care, thereby improving short-term mental health outcomes and reducing subsequent emergency callouts [18, 19]. Additionally, the incorporation of mental health expertise enables more effective de-escalation techniques, preventing the escalation of crises, with co-response teams better able to calm situations compared to police alone [19]. Importantly, young people and their families have more positive perceptions of co-response interventions [28], which contrast sharply with the fear and distrust often associated with police-only interventions.

Integrated models support the transfer of knowledge and skills across professional and organisational boundaries, allowing for comprehensive care [29]. This interdisciplinary approach ensures that all aspects of a young person’s crisis, including medical, psychological, and safety concerns, are addressed. Joint training sessions and shared response protocols enhance the effectiveness of co-response teams [30]. Regular training for all team members on mental health issues and crisis intervention strategies can enhance the likelihood of successful outcomes [18]. Additionally, engaging with community stakeholders, including young people and their families, to design and implement co-response models increases their acceptance and efficacy. Stakeholder perceptions and feedback should be integral to shaping a co-response, ensuring that the model meets the specific needs of the community it serves [31]. In terms of resources, a significant role in the success of co-response models is sufficient funding and staffing to ensure that co-response teams can operate effectively and respond to crises promptly [32]. Finally, policy frameworks that support collaboration between police and mental health services are crucial for the success and sustainability of co-response initiatives [33]. In summary, factors contributing to effective co-response models in the international literature include interdisciplinary collaboration, training and joint response protocols, community and stakeholder engagement, resource availability, and policy and legislative support. Effective communication and collaboration between police, mental health professionals, and paramedics are critical.

To conclude, co-response models could offer substantial benefits for young people experiencing mental health crises by improving safety, enhancing mental health outcomes, and fostering positive perceptions between service users and first responders. Effective implementation relies on interdisciplinary collaboration, adequate training, stakeholder engagement, resource availability, and supportive policies. The international literature suggests co-response models can be beneficial, with local planning and stakeholder engagement considered necessary to inform adaptations to local contexts to address the complex needs of young people in crisis in their locality. Therefore, a realist synthesis drawing upon international and national literature, UK service level documents, alongside interpretation and translation workshops with stakeholders, could meaningfully inform the development of a co-response in a UK context for children and young people experiencing a mental health crisis. A realist review is particularly suited to examining how and why first responders’ interventions can impact young people experiencing a mental health crisis, given the complexity and variability of care in this area. Realist methods are recommended for researching complex interventions, as they go beyond identifying whether an intervention works to explore the mechanisms, contexts, and conditions that influence outcomes [34]. Concerns around inconsistent care quality, the potential for coercion, and the emotional demands placed on first responders [12, 13, 16] highlight the need for a nuanced understanding of what works, for whom, and under what circumstances. A realist approach can provide valuable insights to inform policy and training, ensuring that emergency responses are compassionate, evidence based, and minimise unintended negative consequences. Realist synthesis is an increasingly used approach that generates an understanding of the full range of impacts (intended and unintended) and how, why, and under what circumstances these occur, which is essential to guiding service delivery. However, the methodology is still evolving, and, therefore, publishing detailed protocols is important to guide methodological development as well as providing transparency around the research process for this study.

### Aim

It is to develop a programme theory of the underlying generative mechanisms by which, and contexts within which, an integrated joint response between a police officer and mental health practitioner impacts shared decision-making, role clarity, mental health outcomes, use of restrictive practices, and experiential outcomes for young people experiencing a first response to a mental health crisis.

### Registration of protocol

The protocol was registered on PROSPERO on 29 May 2024 (CRD42024542081), and the review will be conducted and reported in adherence to RAMESES quality and publication standards [35].

Building upon recent guidance [36], the review aims to enhance relevance by prioritising evidence that contributes to understanding generative causation, not just methodological quality. To promote richness, sources will be selected that provide detailed insights into mechanisms, contexts, and outcomes, ensuring transparency in how evidence is appraised and selected, detailing the criteria used.

## Method and analysis

### Realist review

A realist review seeks to understand how and why interventions work ((or do not work) in different contexts, using the heuristic context + mechanism = outcome (C + M = O; [37]) to explain causation. Unlike traditional systematic reviews, which focus on assessing the effectiveness of interventions through aggregating evidence, realist reviews take a theory-driven, interpretative approach to synthesising evidence from diverse sources, including published studies, policy documents, and grey literature [38]. This approach recognises that interventions may be effective in some contexts but not others, for some people but not others, making it particularly useful for complex social and health interventions such as emergency health responses. The realist review begins with initial programme theories that outline our current understanding of emergency responses to young people experiencing mental health crises, which will be iteratively refined as evidence is synthesised [39].

The theory will be developed to explain the underlying mechanisms generating the full range of impacts (intended and unintended) of joint emergency responses for young people and their families. The theory will identify key contextual factors that trigger these underlying causal mechanisms; thus, it is likely to be transferable to similar interventions in different settings with appropriate contextual adaptations. As highlighted in the MRC guidance, developing a robust programme theory enhances the inter-setting transferability of interventions and supports the generation of evidence that is valuable to decision-makers [40]. This theoretical framework can inform the design and implementation of complex co-response interventions for mental health crises in diverse and dynamic environments. To develop our programme theory, two sources will be drawn on — data from documents included in our review and anonymised and unattributed data from interpretation workshops with stakeholders. The combined interpretation process with members of the research team and stakeholders will ensure the review benefits from diverse perspectives. This testing involves iterative processes, such as abductive reasoning and retroduction, leading to the development of a better refined realist programme theory [39].

### Research question

The overarching primary research question guiding this review is as follows: *How can emergency co-responses for young people (aged 25 and under) experiencing mental health crises reduce the need for emergency hospitalisation and restrictive practices?*

Secondary questions include the following:

1. How, why, for whom, in what contexts, and to what extent can first responders to mental health crises support young people and their families at home?
2. What works in terms of co-responding and the collaboration of first responders from different services when responding to young people experiencing mental health crises? The review focuses specifically on police-mental health collaboration; the programme theory could offer helpful clues for wider integrated and co-response approaches for young people, considering both intended and unintended consequences of responders from specific workforces.
3. How can the integration of services benefit young people experiencing mental health crises and their families?

This realist review will identify how, why, for whom, in what contexts, and to what extent first responders to mental health crises can support young people and families. A brief exploratory scoping search conducted by an NHS knowledge exchange specialist revealed the literature is heterogeneous with a wide array of terminology and service delivery models used internationally to respond to youth mental health crises. All have implicit programme theories underlying their design, but there is no clear programme theory guiding this approach. Hence, a realist review is appropriate. The search strategy has been developed with specialist librarians, guided by the project team, and in consultation with stakeholders (e.g. youth and public advisors, service providers, and researchers working in the field, such as the CAMH-Crisis team [41]). The strategy was developed for MEDLINE and will be translated for other major databases. Pawson et al.’s (2005) proposed method for conducting realist reviews will be employed, which allows for interpretation and customisation, helpful for synthesising the heterogeneous literature that will inform this review [37]. The five adapted steps from Pawson et al. (2005) will be as follows: (1) Define the review scope, (2) develop initial programme theories, (3) search for evidence, (4) select and appraise evidence, and (5) extract and synthesise data [26].

Steps 4 and 5 will be further supported through interpretation workshops with the research team and stakeholders. For this review, stakeholders refer to police officers and health and social care practitioners with experience of engagement with crisis services. The process is iterative, and steps may overlap or proceed in parallel as appropriate [39] or be revisited as the review progresses.

#### Step 1: define the review scope

Preliminary exploratory literature searches identified existing research on co-responses to young people in a range of initiatives (e.g. street triage [32]) and suggest co-responses between services lead to better outcomes for young people. Our preliminary searches have helped us to determine the size of the available literature base, which has confirmed there is sufficient literature specifically attending to children, young people, and young adults to include only literature focused on an under 25-year age group. Preliminary searches and discussions with our regional and national stakeholders have helped us to identify relevant additional search terms [8].

Questions arising from initial familiarisation with the relevant literature identified include the following:

1. What qualities, skills, and behaviours in first responders are beneficial for young people experiencing a mental health crisis and their families across contexts?
2. What are the barriers and facilitators to emergency responses perceived as helpful to young people experiencing a mental health crisis and their families and in what contexts are barriers and facilitators present?
3. What are the intended and unintended consequences for young people experiencing a mental health crisis and their families encountered through co-responder models of emergency care?
4. What are the mechanisms by which co-responder models of emergency care improve outcomes for young people in crisis and their families?
5. What are the contexts that influence mechanisms involved in emergency responses for mental health for young people in crisis and their families?

The context-mechanism-outcome (CMO) configuration will be used for this review. Within this review, context refers to the conditions that function to influence outcomes (via mechanisms), such as the configuration of services that support families to support their child through a mental health crisis at home, preventing the need for emergency department admissions and mental health hospital admissions. Further examples include social determinants and pre-existing associations with or relationships between young people, families, police officers, and NHS practitioners [42]. Context can trigger or modify a mechanism [43]. A mechanism is the hidden context-sensitive causal force activated in specific contexts, leading to outcomes [44]. Outcomes are the intended or unintended consequences of the intervention, arising from the interaction between context and mechanism [45]. Examples might include the learning of new coping skills by young people and carers, collaborative risk formulations and safety plans, or changes in perceptions and relationships between families and police officers.

#### Step 2: develop initial programme theories

Initial programme theories describe how interventions are assumed to cause outcomes. Often, but not always, causation in initial programme theories may contain context-mechanism-outcome configurations (CMOCs; [46]). The initial programme theories (IPT) were developed through familiarisation with the literature reviewed to develop the research study and stakeholder conversations, which gradually informed the development of preliminary theories through perspective taking and consensus building around key concepts and issues. An example of an initial programme theory developed from the familiarisation process is as follows.

A joint response model of care (JRM) for children and young people experiencing a mental health crisis integrates mental health practitioners, social workers, and police officers to provide a fast, coordinated, compassionate response. This approach can ensure comprehensive care by addressing both immediate crises and underlying issues while also enhancing safety and reducing restrictive practices. Early intervention prevents escalation, and resource efficiency is improved through interagency collaboration, reducing duplication and enhancing cost-effectiveness. Training and shared expertise strengthen professional capacity, ensuring a more skilled workforce. A joint response also builds community trust, encouraging families to seek support. Family involvement is central, with psychoeducation helping caregivers navigate mental health services. Developmentally appropriate communication, cultural sensitivity, and tailored de-escalation techniques support neurodiverse and vulnerable CYP. By integrating expertise from different sectors, the JRM could foster a holistic approach that improves outcomes, reduces hospital admissions, and ensures timely, effective crisis intervention.

Where possible and relevant, substantive theories relevant to each level of analysis will be selected: individual (micro), interpersonal (meso), and organisational (macro). Selection will be guided by the extent to which each theory addresses our research aims and their explanatory value, i.e. to what extent they help to identify generative causal explanations for key outcomes. This approach ensures that our theoretical framework is both purposeful and rigorous, enhancing the depth and clarity of our analysis. Prioritising theories with strong explanatory value maximises their ability to reveal underlying causal mechanisms, leading to more meaningful insights. To ensure that programme theories are relevant to service providers and users, the review will use the MoSCoW co-production method with stakeholder groups. This approach will allow stakeholders to actively participate in theory prioritisation and selection, ensuring their perspectives shape the final framework [47].

Building upon the recent work of the CAMH-Crisis team [41], findings will be recorded specifically in relation to how emergency services and first responders can do the following: (1) where appropriate, keep young people in their home environment rather than hospital; (2) assess needs and establish support plans; (3) improve family engagement with community treatment, where possible and appropriate; (4) create links between young people, families, and community mental health support; (5) offer opportunities for peer support; (6) manage the present crisis; (7) assess risk and consider referrals to suitable children’s services; and (8) train and/or supervise staff. A list of priorities will also be discussed with the stakeholder groups to gain a broader perspective upon key points for consideration within the synthesis.

#### Step 3: evidence search

Multiple databases will be searched: Embase, CINAHL, Social Policy and Practice, MEDLINE, PsycINFO, and AMED to identify relevant publications (see supplementary material 1 for example of search strategy). The inclusion criteria will focus on studies relevant to developing or testing programme theories.

To ensure inclusion of relevant publications and documents, the following tasks will be undertaken to refine the selection of included documents:

- Cross-check reference lists from selected primary and secondary studies (snowballing).
- Citation searches on Scopus and Google Scholar (lateral searching).
- Seek input from colleagues and stakeholders to identify other relevant publications, guidelines, or policies.
- Discuss emerging findings with regional and national steering committees to discuss whether the scope needs to be broadened or narrowed at key stages of the process.

##### Screening process

Database searches will be imported into Rayyan [48], where de-duplication will occur using the tools sensitive software to identify duplicates. During the screening of identified documents, our initial inclusion criteria will be broad to capture all study designs and nonempirical documents [49]. Two members of the team (L. O., A. A.) will review documents using Rayyan and select the manuscripts for inclusion, agreeing by consensus where there is uncertainty, with further discussion with members of the research team (S. P., Z. E., G. W., F. L.) where necessary. A random 10% of the articles will be double screened by PG to confirm the accuracy of the decision-making process, with the wider study team being available for any discrepancies in decisions [50]. The inclusion criteria are as follows:

- *Focus*: Documents on emergency responses for young people experiencing a mental health crisis
- *Study design*: All study designs
- *Non-empirical data*: Opinion/commentary pieces that contribute to theory development
- *Settings*: Inpatient, outpatient, emergency triage, or home-based care settings
- *Participants*: All young people (up to and including 25 years old), parents/carers reflecting upon emergency responses for their child, and practitioner accounts of being a first responder. As this review is exploratory, underserved groups such as neurodiverse young people, different ethnic minorities, LGBTQIA + individuals, and people in remote or rural areas will be identified and further informed by the existing literature.
- *Interventions*: Any emergency response for young people experiencing a mental health crisis (and/or informal parents/carers), operationalised through co-responses between mental health services and law enforcement agencies, such as police officers working alongside mental health practitioners
- *Outcome measures*: Instances of restrictive practices (e.g. Section 136), emergency admissions to hospital, the impact of responses/responders upon relationships between responders, young people, and families. Section 136 of the Mental Health Act (1983) in England and Wales grants police officers the authority to detain an individual in a public place if they appear to be experiencing a mental health crisis and are deemed in need of immediate care or control. The individual can then be taken to a designated health-based place of safety or, in some cases, a police station for assessment by mental health professionals. This provision is intended to ensure that people in crisis receive appropriate care rather than being placed in the criminal justice system. Similar laws exist in other countries, but the specifics of implementation and available crisis support services may vary.

The inclusion and exclusion criteria will likely evolve as the review progresses, adapting to the developing programme theory. Given the expected high volume of relevant literature, additional criteria may be introduced to refine the focus of the review and prioritise the aspects of the programme theory most important to stakeholders or most actionable in emergency response settings.

#### Step 4: selection and appraisal of evidence

Included full-text documents will then be assessed based on the criteria of relevance and richness. Studies will be assessed for relevance (i.e. whether the document tells us anything about the mechanism and contexts of interest) and richness. Assessments of richness will focus on those documents that make significant contributions to CMOCs and/or programme theory. Richness will also be assessed at the level of the programme theory [51].

##### Initial sorting and analysis

Initial sorting of all identified materials will begin using criteria aimed at pinpointing those most likely to contain pertinent data for developing the programme theory and context, mechanism, and outcome configuration (CMOC; [49]). The project team and stakeholder groups’ content expertise will guide the selection of materials for initial analysis. A narrower focus will facilitate the initial stages of programme theory and CMOC development, which is why the initial focus will be on emergency responses specifically for young people experiencing a mental health crisis. As gaps in our programme theory or CMOCs emerge, additional documents previously deemed lower priority will be reviewed to find further relevant data and continue to review new publications and associated searches, as necessary. This gradual expansion will help manage the large volume of diverse materials effectively.

#### Step 5: data extraction and synthesis

Data will be extracted using a bespoke form and code relevant text (see supplementary material 2). The data will be synthesised to identify patterns in contexts, mechanisms, and outcomes. Where necessary and appropriate, we will link findings to other theories (e.g. substantive theories) to explain emerging patterns.

Data extraction will focus on extracting explanatory accounts and mapping what these data are about (e.g. details of the context, nature of the intervention). Relevant literature will be uploaded to NVivo [52], and information relating to important concepts and contexts, mechanisms, and outcomes will be coded. Codes will be inductive (generated to categorise data in the included literature), deductive (identified prior to data extraction and informed by the initial programme theory), and retroductive (inferences about what might be functioning as mechanisms for outcomes of interest within the programme theory; [53]). Study characteristics will be tabulated through the extraction process, with context details of each data source recorded in a data extraction database in Excel. Data extraction will focus upon extracting key elements from each study, including the study’s setting, population, co-response intervention details, and reported outcomes, as well as contextual factors and underlying mechanisms. The characteristics of studies table will include columns for the following elements: Authors and year of publication, study design, setting (including country/countries), population, intervention components, stage of intervention (e.g. feasibility, trial, established treatment), context, mechanisms, outcomes, and key findings.

**Fig. 1:**
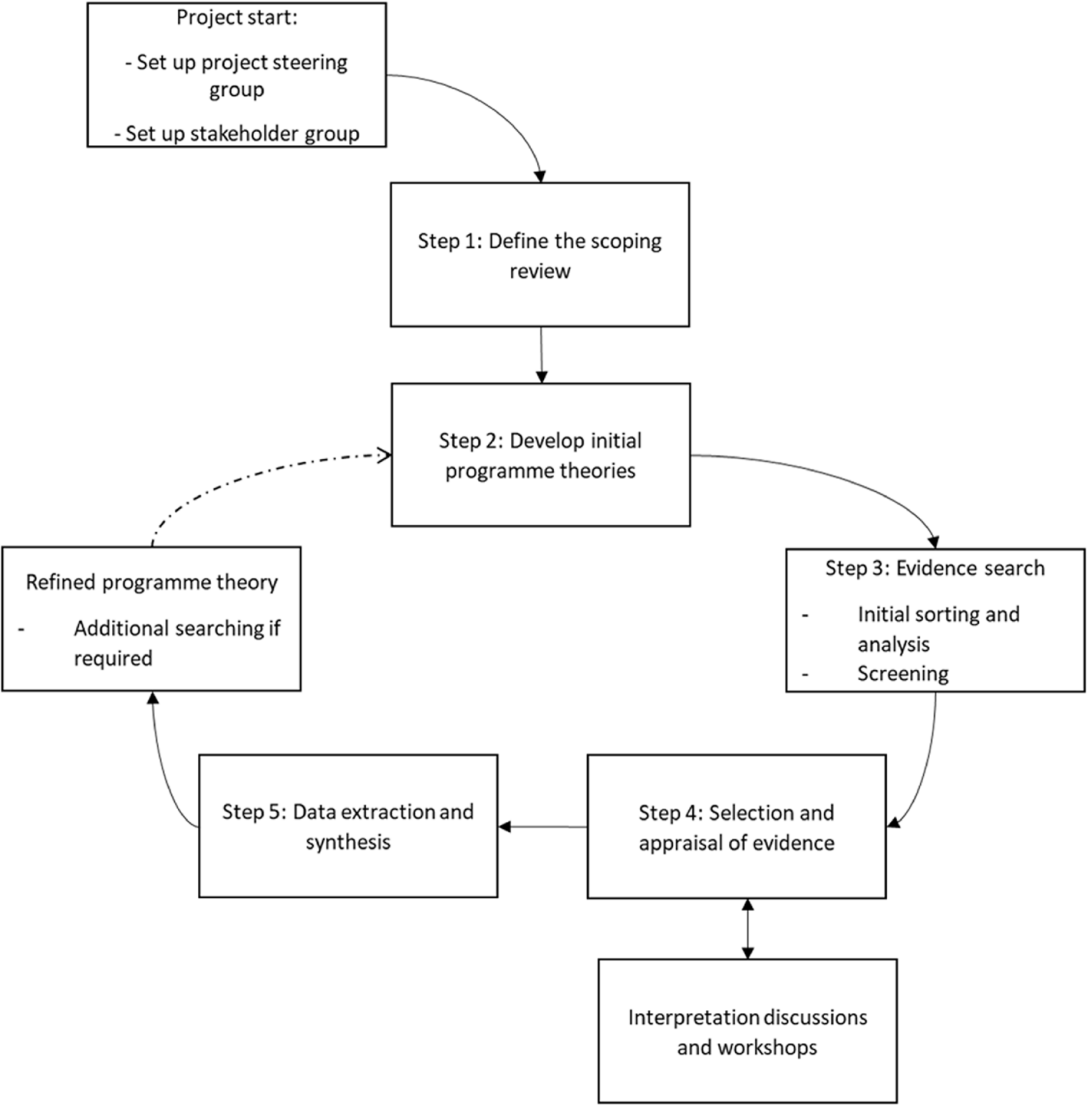
Flow diagram of realist review methodology

This structure will facilitate the systematic organisation of complex data on complex co-response interventions, allowing for the identification of patterns and insights crucial for a realist synthesis. Following this approach will ensure comprehensive and coherent data extraction and presentation [54, 55], supporting the robust synthesis of findings. In realist research, ‘context’ has two distinct interpretations: (a) the literal backdrop within which events take place, such as policy and social factors, and (b) the relational, emergent, and dynamic forces that interact with mechanism, including cultural norms and relationships [43]. This distinction is important, as it acknowledges the different ways in which context can shape outcomes. For instance, when exploring co-response interventions, the team will identify contextual factors that include both the physical and the relational contexts. These factors might include geographical settings, institutional environments, and specific population characteristics (e.g. demographic information, socio-economic status), all of which influence how co-response interventions unfold and their resulting outcomes (Fig. 1).

The underlying processes and participant responses that drive the observed effects will also be explored, thereby explaining how and why certain outcomes are achieved in specific contexts. Finally, detailed outcome reporting of the primary outcomes and any unintended consequences or secondary outcomes will support our developing understanding of the impact of co-response interventions and in identifying patterns across different studies [56].

Analysis will employ a realist logic of analysis, which will involve building causal explanations for outcomes that are expressed as context-mechanism-outcome configurations (CMOCs). The coded data will be drawn upon to identify outcomes of importance within the programme theory. For each of these outcomes, analysis will work ‘backwards’ from the outcome of interest and use retroductive reasoning to infer what might be functioning as the mechanism for the outcome. Inferences from relevant data will be made regarding what is functioning to trigger the mechanism — i.e. what is functioning as context. Using this process, the CMOCs within the programme theory will be developed. Data will be drawn on from across individual documents and integrate and consolidate explanatory accounts. Particular attention will be paid to accounts of potential negative impacts and those that are unexpected, contradictory, or challenge our initial programme theories. Throughout the development of these initial theories, data will be prioritised that can inform the development and operationalisation of a co-response between the police and mental health practitioner for young people experiencing a mental health crisis. Less relevant working theories will be noted in a separate document for review later in the process to offer points for critical reflection. A final stage of interpretation workshops or individual discussions, depending upon the preference of the participants (*N* = 10), will take place with people aged 18 years and over. Participants will have their own direct connection to emergency responses for mental health (e.g. young adults with lived experience, parents, carers, and first responders), leading to a refined middle-range programme theory. A minimum age limit of 18 has been set, as discussing the review’s findings in relation to lived experience could be distressing for a young person who has recently been through their own mental health crisis. While it is crucial to involve young people in the review, it is equally important to prioritise their well-being and consider how recently they have experienced similar challenges.

### Interpretation discussions and workshops

The purpose of using interpretation workshops and discussions with professional stakeholders during a realist synthesis is to develop initial programme theories by incorporating diverse perspectives and expertise. This approach ensures that the synthesis is grounded in real-world contexts and is more likely to produce actionable insights. Through our existing professional networks, 20 relevant stakeholders, including healthcare professionals, commissioners, police officers, and other non-researcher contributors, will be identified. Stakeholders will have a direct interest or expertise in relation to responding to mental health crises. Stakeholders will have the option of discussing their perspective individually with a member of the review team or joining an online discussion forum or workshop.

In preparation for the discussions, summaries of relevant literature and initial programme theories will be provided. A clear agenda and objectives will be set. Discussions will be held with a focus on open dialogue and collaborative exploration of the literature. Stakeholders will be encouraged to share their experiences and insights, highlighting practical implications and contextual factors. A semi-structured discussion guide will ensure consistency while allowing flexibility for emergent topics. Discussions will be recorded and transcribed for transparency, record-keeping, and thorough analysis. This process will then be repeated with up to 10 people with their own lived experience of emergency responses for mental health crises. The same realist logic of analysis as outlined in Step 5 above will be used to help further develop and test (confirm, refute, or refine) the CMOCs and programme theory developed from the literature. Iteratively, the team will refine initial programme theories based on the analysis, integrating stakeholder feedback. Preliminary findings and revised programme theories will be shared with stakeholders for validation. Feedback will then be incorporated to further refine the theories, ensuring they accurately reflect the collective insights and are grounded in real-world contexts. Documentation of the process and outcomes of the workshops and discussions will occur, including how stakeholder input influenced the development of programme theories. This approach will enhance the relevance and applicability of the realist synthesis, ensure a comprehensive understanding of complex interventions and their contexts, and promote stakeholder buy-in and the implementation of findings [57]. The evolving programme theory will be shared at regular intervals with the research team and stakeholder groups in written and diagrammatic form for ongoing refinement, integration, and prioritisation of aspects to take forward for testing in a realist evaluation that will follow on from this realist review.

#### Dissemination

We are committed to ensuring high quality and high impact dissemination of findings from our research study aimed at improving emergency and crisis mental healthcare for young people. To achieve this, there will be collaboration with stakeholders to develop tailored outputs for specific audiences from the review. In addition to policy-level dissemination, we will share information via mainstream and social media platforms to reach a broader audience. Press releases and media briefings will be organised to highlight key findings and their implications. Social media campaigns will be designed to engage the public, share insights, and generate discussions. Consistent contact points with national policymakers will provide avenues for broader dissemination with Integrated Care Boards (ICBs) nationally, clinical networks, and Mental Health Trust Executives through established NHSE forums.

Community events will be organised to present our findings directly to young people, their families, and local service providers. These events will include workshops, seminars, and interactive sessions to facilitate a reflective dialogue and ensure that the community’s voice is heard and integrated into our work. The team will develop accessible summaries and infographics to ensure that complex information is understandable and actionable for all stakeholders. There will be opportunities to share regular updates with the Department of Health and Social Care (DHSC) and NHS England, College of Policing, and the Home Office. Through this comprehensive dissemination strategy, we aim to ensure that our findings inform and influence policy and practice at local, regional, and national levels, ultimately improving emergency and crisis mental healthcare for young people.

## Discussion

This research seeks to enhance crisis response services for young people experiencing mental health emergencies by identifying ways to improve collaboration between emergency responders. With rising mental health challenges among children and young people, there remains a significant gap in understanding and optimising emergency care pathways. Police data highlights an increase in crisis-related emergency calls, yet officers often lack the specialised training needed to provide effective support. Meanwhile, mental health practitioners possess the necessary expertise, but children’s mental health services are typically unavailable during evenings and weekends, particularly in rural areas. This realist synthesis aims to develop a programme theory that identifies the mechanisms and conditions under which police officers and mental health practitioners can work together more effectively. By informing future service improvements, the findings will help ensure that young people in crisis receive timely and appropriate support.

The methodology for this realist review includes a five-step process: defining the scope, developing initial programme theories, conducting evidence searches, selecting and appraising evidence, and synthesising data. Comprehensive database searches will be complemented by systematic searches, citation tracking, grey literature review, and interpretation workshops. Data selection will prioritise relevance and richness, focusing on establishing causal relationships between contexts, mechanisms, and outcomes. Dissemination strategies will include conference presentations, peer-reviewed journal articles, and collaborations with healthcare and police organisations, ensuring the findings are accessible to a wide range of stakeholders and can inform future policies and practices.

## Supplementary Information

Supplementary Material 1. Medline search strategy.

Supplementary Material 2. Characteristics of included studies.

## Data Availability

All data generated or analysed during this study are included in this published article (and its supplementary information files).

## Abbreviations

RAMESES: Realist and Meta-narrative Evidence Syntheses: Evolving Standards
NHS: National Health Service
GMP: Greater Manchester Police
UK: United Kingdom
CMO: Context-mechanism-outcome
CMOCs: Context-mechanism-outcome configurations
ICBs: Integrated Care Boards
DHSC: Department of Health and Social Care
