# Supplementary material for: Realist review protocol for understanding young people’s experiences of engaging with police-mental health practitioner collaboration in emergency responses to mental health crises
Source: Syst Rev. 2025 Sep 24;14:173. doi: 10.1186/s13643-025-02882-4 (PMC12462277; doi:10.1186/s13643-025-02882-4)
Supplement: Supplementary file 1 — Supplementary Material 1. Medline search strategy. [file 13643_2025_2882_MOESM1_ESM.docx]

Ovid MEDLINE(R) ALL <1946 to May 21, 2024>

1 exp Adolescent/ 2248288

2 exp Child/ 2205872

3 exp Young Adult/ 1033041

4 exp Students/ 176080

5 adolescen*.tw. 367084

6 teen*.tw. 36439

7 child*.tw. 1693153

8 "young person".tw. 1401

9 "young adult*".tw. 126882

10 "young people".tw. 38830

11 "young patient*".tw. 36556

12 youth*.tw. 103370

13 Juvenile*.tw. 97089

14 p?ediatric*.tw. 471483

15 student*.tw. 382573

16 pupil*.tw. 35443

17 "young offender*".tw. 537

18 1 or 2 or 3 or 4 or 5 or 6 or 7 or 8 or 9 or 10 or 11 or 12 or 13 or 14 or 15 or 16 or 17 4963633

19 exp Crisis Intervention/ 6327

20 crisis.tw. 84263

21 crises.tw. 18921

22 "rapid response".tw. 9054

23 19 or 20 or 21 or 22 110820

24 exp Mental Health/ 67363

25 exp Mental Disorders/ 1490579

26 exp Psychology, Adolescent/ 13843

27 exp Psychology, Child/ 13588

28 exp Adolescent Psychiatry/ 3132

29 exp Child Psychiatry/ 5839

30 exp Community Psychiatry/ 2091

31 exp Mental Health Services/ 107451

32 exp Community Mental Health Centers/ 3337

33 mental*.tw. 496940

34 psych*.tw. 1038036

35 camhs.tw. 670

36 exp Suicide/ 77244

37 exp Self-Injurious Behavior/ 86431

38 suicid*.tw. 100390

39 "self-harm".tw. 8362

40 "self-injur*".tw. 6708

41 24 or 25 or 26 or 27 or 28 or 29 or 30 or 31 or 32 or 33 or 34 or 35 or 36 or 37 or 38 or 39 or 40 2483094

42 18 and 23 and 41 8213

43 (mental adj5 emergenc*).tw. 2486

44 (psych* adj5 emergenc*).tw. 6534

45 (mental adj3 "critical incident*").tw. 7

46 (psych* adj3 "critical incident*").tw. 35

47 (mental adj3 urgent).tw. 126

48 (psych* adj3 urgent).tw. 214

49 (mental adj3 distress).tw. 4528

50 (psych* adj3 distress).ti. 8565

51 43 or 44 or 45 or 46 or 47 or 48 or 49 or 50 21752

52 18 and 51 7981

53 42 or 52 15663

54 Police/ 7049

55 Emergency Responders/ 1263

56 Law Enforcement/ 4636

57 (police or policing).tw. 19661

58 (inter-agency or interagency or multi-agency or multiagency).tw. 4347

59 (co-responder or coresponder or co-response or coresponse).tw. 136

60 (inter-professional or interprofessional).tw. 17531

61 intersectoral collaboration/ 2617

62 Cooperative Behavior/ 46392

63 Triage/ 15427

64 street triage.tw. 20

65 (hotline* or helpline*).tw. 2750

66 Hotlines/ 2986

67 ("999" or "911").tw. 34367

68 mobile.tw. 134727

69 blue light.tw. 12565

70 (de-escalat* or deescalat*).tw. 5199

71 "brief intervention*".tw. 5167

72 "collaborat*".tw. 215306

73 (joint response or joint or joined or joined-up).tw. 358849

74 54 or 55 or 56 or 57 or 58 or 59 or 60 or 61 or 62 or 63 or 64 or 65 or 66 or 67 or 68 or 69 or 70 or 71 or 72 or 73 839797

75 42 and 74 857

76 52 and 74 418

77 53 and 74 1183
