# Supplementary material for: Realist review protocol for understanding young people’s experiences of engaging with police-mental health practitioner collaboration in emergency responses to mental health crises
Source: Syst Rev. 2025 Sep 24;14:173. doi: 10.1186/s13643-025-02882-4 (PMC12462277; doi:10.1186/s13643-025-02882-4)
Supplement: Supplementary file 2 — Supplementary Material 2. Characteristics of included studies. [file 13643_2025_2882_MOESM2_ESM.docx]

| **Characteristics of included studies** | | | | | | | | | | | | |  |
| --- | --- | --- | --- | --- | --- | --- | --- | --- | --- | --- | --- | --- | --- |
| **Authors** | **Title** | **Year** | **Country/ countries of origin** | **Type of report** | **Aim(s)** | **Study design** | **Sample size and setting** | **Participant characteristics** | **Intervention/ model/ service** | **Methods of data collection** | **Methods of data analysis** | **Key Findings** | **Recommendations** |
|  |  |  |  |  |  |  |  |  |  |  |  |  |  |
|  |  |  |  |  |  |  |  |  |  |  |  |  |  |
